# Retrospective analysis of the apnea test and ancillary test in determining brain death

**DOI:** 10.5935/0103-507X.20200069

**Published:** 2020

**Authors:** Halil Erkan Sayan

**Affiliations:** 1 Department of Anesthesiology and Reanimation, Bursa Yuksek Ihtisas Training and Research Hospital, University of Health Sciences - Bursa, Turkey.

**Keywords:** Brain death/diagnosis, Apnea, Tissue and organ procurement, Morte cerebral/diagnóstico, Apneia, Obtenção de órgãos e tecidos

## Abstract

**Objective:**

We investigated the frequency of apnea tests, and the use of ancillary tests in the diagnosis of brain death in our hospital, as well as the reasons for not being able to perform apnea testing and the reasons for using ancillary tests.

**Methods:**

In this retrospective study, the files of patients diagnosed with brain death between 2012 - 2018 were examined. The preferred test was determined if an ancillary test was performed in the diagnosis of brain death. The rate and frequency of use of these tests were analyzed.

**Results:**

During the diagnosis of brain death, an apnea test was performed on 104 (61.5%) patients and was not or could not be performed on 65 (38.5%) patients. Ancillary tests were performed on 139 (82.8%) of the patients. The most common ancillary test was computed tomography angiography (79 patients, 46.7%). Approval for organ donation was received in the meetings with the family following the diagnosis of brain death for 55 (32.5%) of the 169 patients.

**Conclusion:**

We found an increase in the rate of incomplete apnea tests and concordantly, an increase in the use of ancillary tests in recent years. Ancillary tests should be performed on patients when there is difficulty in reaching a decision of brain death, but it should not be forgotten that there is no worldwide consensus on the use of ancillary tests.

## Abstract (pt)

**Objetivo:**

Investigamos a frequência dos testes de apneia e o uso de exames complementares para o diagnóstico de morte cerebral em nosso hospital, assim como as razões para a não realização do teste de apneia e para utilização de exames complementares.

**Métodos:**

Neste estudo retrospectivo, examinaram-se os arquivos de pacientes com diagnóstico de morte cerebral entre 2012 e 2018. O exame preferido foi determinado quando um exame complementar foi realizado para o diagnóstico de morte cerebral. Analisaram-se a taxa e a frequência de uso desses exames.

**Resultados:**

Durante o diagnóstico de morte cerebral, o teste de apneia foi realizado em 104 (61,5%) pacientes, e não foi ou não pôde ser realizado em 65 (38,5%) deles. Realizaram-se exames complementares em 139 (82,8%) pacientes. O exame complementar mais comumente utilizado foi a angiografia por tomografia computadorizada (79 pacientes, 46,7%). Foi recebida aprovação para doação de órgãos nas reuniões com familiares após o diagnóstico de morte cerebral para 55 (32,5%) dos 169 pacientes.

**Conclusão:**

Nos anos mais recentes, identificamos aumento na taxa de testes de apneia incompletos e, concordantemente, elevação no uso de exames complementares. Os exames complementares devem ser utilizados nos pacientes quando há dificuldade para chegar à decisão do diagnóstico de morte cerebral, mas não se deve esquecer que não existe um consenso mundial a respeito do uso de exames complementares.

## INTRODUCTION

Brain death, which is defined as the irreversible loss of all brain functions including the brain stem, was first defined in 1968 at Harvard University.^(1)^ The apnea test, which indicates the absence of spontaneous respirations following the occurrence of coma and the absence of 7 brainstem reflexes, is one of the basic tests for the clinical determination of brain death.^(2)^ Although brain death has been described as a clinical diagnosis, some authors advocate the routine use of ancillary tests along with clinical examination to determine brain death.^(3)^ Some authors suggest that ancillary tests are unnecessary and that when brain death diagnosis cannot be made based on clinical examination, brain death diagnosis should not be considered.^(4)^

Today, the general practice in the diagnosis of brain death is to support the diagnosis with ancillary tests if there is uncertainty in the clinical examination and/or if the apnea test cannot be performed.^(4)^ Ancillary tests are based on the detection of the absence of blood flow or electrical activity in the brain. Palmer and Bader^(5)^proposed different hypothetical mechanisms for brain death. In the first hypothesis, intracranial pressure (ICP) exceeds the mean arterial pressure - MAP (ICP > MAP), leading to brain and brainstem death. This hypothesis is the idea upon which most ancillary tests are based. In the second hypothesis, ICP does not exceed MAP; therefore, cerebral blood flow is maintained. However, this leads to neuronal and axonal damage and eventually results in brain death. It is stated that this second mechanical hypothesis constitutes the basis of the mechanism that may cause a false negative interpretation of blood flow.^(6)^

Ancillary tests based on the assessment of blood flow are based on the hypothesis that in the absence of blood flow to the entire brain, no brain function exists. Therefore, the absence of cerebral blood flow is consistent with the diagnosis of brain death. However, cerebral blood flow tests may give false negative results because ICP decreases in patients who have undergone ventricular drainage or decompressive cranial surgery.^(7)^ Tests evaluating the electrical activity of the brain are based on the assumption that the absence of electrical activity indicates no brain function.^(7)^

Transcranial Doppler ultrasonography (TCD), cerebral scintigraphy, cerebral angiography, magnetic resonance angiography (MRA) and computed tomography angiography (CTA) are used as ancillary tests to assess cerebral blood flow. Electroencephalography (EEG) and somatosensory evoked potentials (SSEP) are commonly known electrical activity tests. The gold standard for ancillary tests is 4-vessel cerebral angiography.^(8)^ In a study examining brain death protocols, it was determined that in 40% of 80 countries, ancillary testing was mandatory along with clinical evaluation.^(9)^ However, no ancillary tests are perfect, and they can give false negative or false positive results.^(10)^ Therefore, research is in progress to assess new tests demonstrating brain tissue oxygenation with the bispectral index scale (BIS), which is a mathematical algorithm of EEG and near infrared spectroscopy (NIRS).^(5,11)^

In this study, we investigated the frequency of the apnea test and the use of ancillary tests in the diagnosis of brain death in our hospital, as well as the reasons for not being able to perform apnea testing and the reasons for using ancillary tests.

## METHODS

In this retrospective study, the files of patients diagnosed with brain death between 2012-2018 were examined after the approval of the local ethics committee.

In our country, 2 ancillary tests are mandatory in newborn to 2-month-old infants, while it is left to the clinician’s discretion to perform ancillary tests in children between 2 months and 18 years of age. However, since clinicians usually tend to perform ancillary tests when the patient at the stage of brain death is a “child”, we thought that the diagnosis of brain death in newborns, infants, and children was a separate subject of research. Therefore, only adult patients over 18 years of age were included in our study.

Demographic data of patients (age, sex), intensive care unit (ICU) admission diagnoses, whether the apnea test could be performed at the diagnosis stage of brain death, and if not, the reason why were examined. The prerequisites accepted for performing the apnea test were core temperature ≥ 36ºC or 97ºF, systolic blood pressure ≥ 100mmHg, eucapnia (partial pressure of oxygen - PaCO_2_ 35 to 45mmHg), absence of hypoxia and euvolemic status. The apnea test was not performed when the patients were under the influence of drugs that may paralyze the respiratory muscles. The donor ratio and the duration from the time of brain death diagnosis to cardiac arrest in nondonor cases were recorded. The preferred test was determined if an ancillary test was performed in the diagnosis of brain death. The rate and frequency of use of these tests were analyzed.

### Statistical analysis

Data were analyzed using Statistical Package for the Social Sciences (SPSS) version 22 (SPSS Inc.; Armonk, NY, USA). The results are presented as numbers and percentages (mean ± standard deviation - SD). Chi-squared or Fisher’s exact test was performed where appropriate. We used one-factor analysis of variance (ANOVA) for comparison when the variables were normally distributed, and the Mann-Whitney U-test when they were not normally distributed. A p value < 0.05 was considered statistically significant.

## RESULTS

Within the study period, 187 patients were diagnosed with brain death. Eighteen of these patients were excluded because they were under 18 years of age. The files of the remaining 169 patients were examined.

Seventy-seven (45.6%) of the patients were female, and 92 (54.4%) were male. The most common ICU admission diagnosis was intracranial hemorrhage (45.6%). This diagnosis was followed by traumatic brain injury (20.7%) and cerebral ischemia (17.8%) (Table 1)

**Table 1:** Demographic data and diagnosis of patients

	All	Donor	Nondonor	p value
Age (F/M), year	56 ± 16.85/54.15 ± 15.99	51.09 ± 16.87/55.12 ± 14.93	57.96 ± 16.58/53.61 ± 16.65	0.414
Sex (F/M)	77/92 (45.6/54.4)	22/33 (40/60)	55/59 (48.2/51.8)	0.315
BMI	25.10 ± 5.18	24.60 ± 5.52	25.30 ± 5.11	0.637
Diagnosis	169 (100)	55 (33)	114 (67)	
Intracranial hemorrhage	77 (45.6)	31 (56.4)	46 (40.4)	0.050
Traumatic brain injury	35 (20.7)	18 (32.7)	17 (14.9)	0.007
Cerebral ischemia	30 (17.8)	3 (5.5)	27 (23.7)	0.004
Brain neoplasms	14 (8.3)	1 (1.8)	13 (11.4)	0.038
Hypoxic brain damage	6 (3.6)	0	6 (5.3)	0.179
Others	7 (4.2)	2 (3.6)	5 (4.4)	1.000

F - female; M - male; BMI - body mass index. Results expressed as mean ± standard deviation or n (%).

After admission to the intensive care unit, brain death testing was started in the first week for 132 (78%) patients, in 1 - 2 weeks for 27 (16%) patients, and in 2 weeks for 10 (6%) patients. The completion period of brain death tests was 1 day for 85 (50%) patients, 2 days for 60 (36%) patients and 3 days or more for 24 (14%) patients. No correlation was found between organ donation decision and the time between ICU admission and completion of brain death tests (p = 0.42).

During the diagnosis of brain death, the apnea test was performed on 104 (61.5%) patients and was not or could not be performed on 65 (38.5%) patients. The test was determined to be positive in all patients who underwent apnea testing. The most common reasons that the apnea test could not be performed or completed was unstable hemodynamics (51 patients, 77.3%), low peripheral oxygen saturation (2 patients, 3%), hypercarbia (1 patient, 1.5%), bradycardia (1 patient, 1.5%), failure to reach the blood gas target (1 patient, 1.5%) and failure to meet the prerequisites for the apnea test (1 patient, 1.5%). For nine patients (13.6%), the reason for not performing the apnea test was not specified.

**Figure 1:**
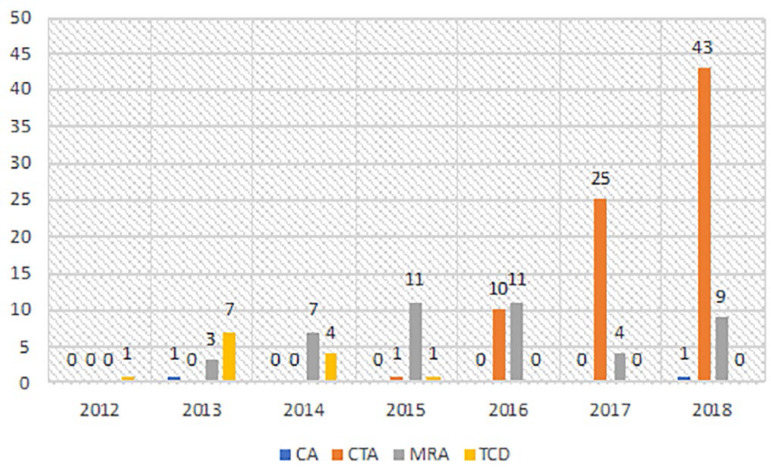
Ancillary tests. CA - cerebral angiography; CTA - computed tomography angiography; MRA - magnetic resonance angiography; TCD - transcranial Doppler ultrasonography.

Ancillary tests were performed on 139 (82.8%) of the patients. It was found that ancillary tests were performed on 74 (71%) of the patients who were positive for the apnea test. The most common ancillary test was CTA (79 patients, 46.7%). This was followed by MRA (45 patients, 26.6%), TCD (13 patients, 7.7%) and cerebral angiography (2 patients, 1.2%). Two patients required a second ancillary test. In the pre-2016 period when there was no CTA in our hospital, TCD was performed on these two patients who could not undergo the apnea test, but upon suspicion, MRA was performed as a second ancillary test. EEG and SSEP were not used for any patient. While the most commonly used ancillary test was MRA until 2016, CTA was performed more often after 2016. Ancillary tests carried out by years are shown in figure 1.

**Figure 2:**
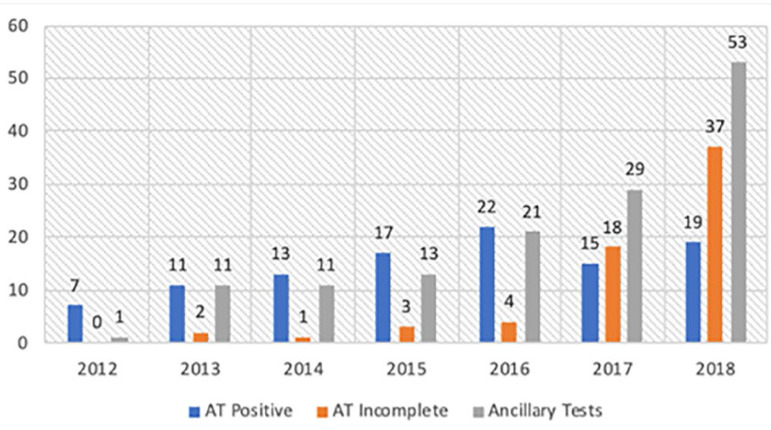
Apnea test and ancillary tests. AT positive - positive apnea test; AT incomplete - incomplete apnea test.

A statistically significant increase was observed in the use of ancillary tests and the number of patients on whom the apnea test could not be performed in 2017 and 2018, compared to 2016 and before (p = 0.001) (Figure 2).

**Figure 3:**
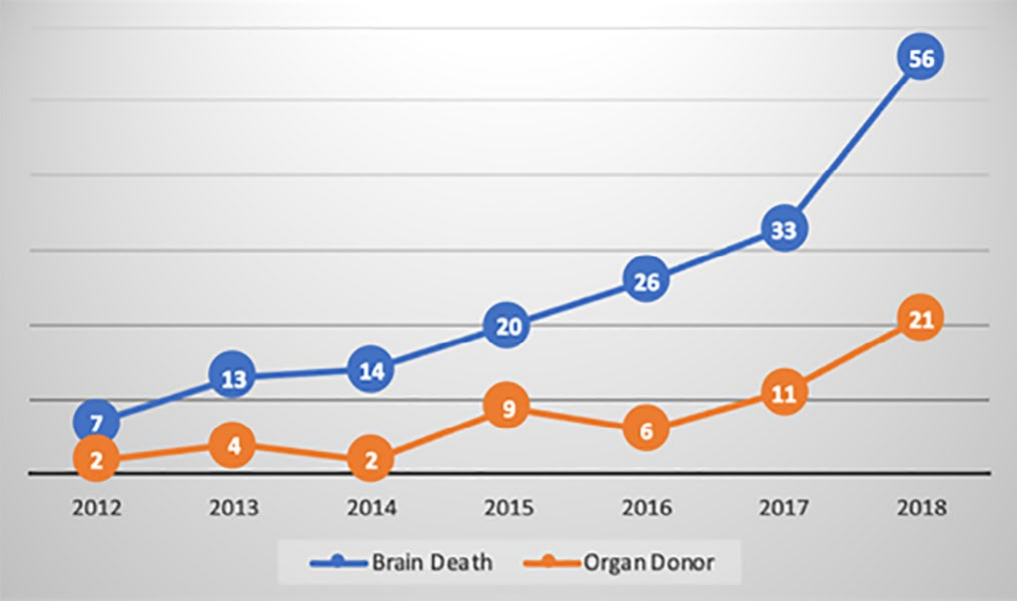
Brain death and organ donor.

Approval for organ donation was received in the meetings with the family following the diagnosis of brain death for 55 (32.5%) out of 169 patients. When the distribution of the diagnosis of brain death by years was analyzed, no increase was detected in the number of donor patients proportional to the diagnosis of brain death (p = 0.32) (Figure 3).

After the diagnosis of brain death, 44 (35%) of 124 nondonor patients were immediately delivered to their families, and the duration until cardiac arrest after the diagnosis of brain death for the remaining 80 patients was determined to be 2.8 ± 0.3 days.

## DISCUSSION

The most important problem surrounding brain death since 1968, when it was first officially defined at Harvard, is how to determine the (irreversible) absence of brain functions. For this purpose, countries have established their own guidelines and set different procedures. “Clinical examination” constitutes the most important part of the diagnosis of brain death. Although brain death is clinically defined, it is preferable to perform ancillary tests, even if it is not mandated by law when there is a suspicion during the diagnosis process, such as persistent effects of sedative drugs, severe facial trauma or failure to perform apnea testing. However, there are significant differences in the use of ancillary tests by country.^(9,12)^ In a study of 226 organ donors in 68 hospitals in the United States, it was detected that approximately a quarter of brain deaths were confirmed by ancillary tests.^(13)^ In our hospital, 82.8% of the patients diagnosed with brain death underwent an ancillary test, and although not compulsory, 71% of the patients who had a positive apnea test had an ancillary test. We think that the reason for resorting to ancillary tests while brain death diagnosis can be made with an apnea test and a clinical examination is to accelerate the waiting period for the second neurological examination that needs to be performed after 12 hours for unstable patients. In the literature, it has been shown that ancillary tests shorten the time to detect brain death.^(14)^ However, it has been reported in the literature that these ancillary tests may delay the diagnosis of brain death and cause damage to the organ donation target by causing confusion (false negative or false positive findings).^(15)^

The apnea test is accepted as the main determinant of brain death. It is essential to the brain death decision in the guidelines of many countries, such as England (1983), France (1996) and the United States (2010).^(16)^ However, in cases such as hypoxia, hypotension, arrhythmia, acidosis and increased intracranial pressure, difficulties are encountered in performing the apnea test.^(17)^ In a study where 228 cases of brain death were examined between 1996 - 2007, the apnea testing rate was found to be 89.9%. In this study, hypotension and hypoxia were detected as the factors that prevented apnea testing.^(18)^ In our study, the apnea test was performed on 61.5% of patients diagnosed with brain death. The most common preventive factor for apnea testing was unstable hemodynamics.

Although apnea testing is defined within specific rules, the lack of training and concerns about the complications of the apnea test limit the use of the test and increase the use of ancillary tests.^(19)^ Studies indicate that ancillary tests should be performed when clinical tests cannot be performed reliably, when prerequisites for performing the test cannot be provided or if they have to be cancelled.^(20)^ In their study on the use of ancillary tests in brain death, Wijdicks et al.^(18)^ found that clinicians turn to ancillary tests mostly when they are in doubt of the results of the apnea test and when the apnea test cannot be completed.^(18)^

The ideal ancillary test should be a non-false positive, easily applicable, and reliable test.^(21)^ The most commonly used ancillary tests in Europe are EEG, SSEP, TCD, perfusion scintigraphy and cerebral angiography. False-positive or more often, false-negative results (patient is clinically diagnosed as brain dead, but ancillary test outputs give contradictory findings) may be obtained with ancillary tests, and this has been reported to be encountered most frequently with EEG.^(8)^ However, in a global survey covering 91 countries in 2015, EEG was found to be mandatory in 28% of the countries and was recommended as an ancillary test in 47%.^(22)^ In a multicenter study conducted in 42 intensive care units in Spain, the most commonly used ancillary tests were stated as EEG (74%) and TCD (37%).^(23)^ Similarly, in a multicenter study in the United States, EEG was identified as the second most preferred ancillary test.^(13)^ In our hospital, we determined that EEG is not preferred as an ancillary test and that all of the preferred ancillary tests are tests showing brain blood flow. We encountered guidelines in the literature stating that EEG is not an appropriate ancillary test in the diagnosis of brain death due to its limitations, and recommending instead ancillary tests showing brain blood flow to be preferred.^(24,25)^

Transcranial doppler ultrasonography is useful for rapidly assessing cerebral blood flow at the bedside, especially in patients with unstable hemodynamics. Monteiro et al.^(26)^ in a meta-analysis consisting of 10 studies found that TCD had a sensitivity of 89% and specificity of 99% compared with clinical examination. Although TCD has been accepted as an ancillary test in our country, we could only use TCD in 13 patients (7.7%) due to the insufficient number of experienced specialists in our hospital.

Among the ancillary tests, cerebral angiography is considered to be the gold standard, and it has been shown to be the most reliable diagnostic method for demonstrating absence of brain circulation.^(27)^ The fact that it is a time-consuming and expensive procedure that cannot be performed at the bedside appears to be the disadvantage of this test.^(28)^ In addition, it has been found that administration of contrast agent can trigger allergic reactions or kidney damage and possibly cause damage by increasing the rejection rate in organ recipients.^(29)^

Recently, the use of CTA, which is a relatively newer test, in brain death diagnosis has become prevalent. Studies have begun to show the reliability of CTA, which indicates the absence of intracranial blood flow.^(29,30)^ Although the data supports the use of CTA as a reliable ancillary test, there is no specific consensus for performing the test or interpreting the results.^(31)^ When the literature is examined, no definite information is found about the limitations of CTA in the diagnosis of brain death.^(32)^ CTA is seen as a promising alternative because it is easy, widely applicable, and noninvasive and can lead to rapid results. Nonetheless, CTA is not considered an ancillary test for the diagnosis of brain death in many countries. It has been reported that cranial defects, decompressive craniectomies and cranial drainage operations can prevent intracranial blood flow from stopping completely, even if the clinical examination is positive for brain death.^(33)^ In our country, the use of CTA as an ancillary test has been accepted, and it was also found to be the most frequently used ancillary test in our study. The main obstacle is that this method is not 100% sensitive for reliable diagnosis. Taylor et al.^(34)^ reported the sensitivity of CTA in the diagnosis of brain death to be 84% in ten studies they found in a Cochrane database search.

In our study, we found an increase in the rate of incomplete apnea tests and concordantly, an increase in the use of ancillary tests in recent years. Since the patient is considered medically and legally dead upon the confirmation of brain death diagnosis, the diagnosis of brain death carries great responsibility. This may explain the significant use of both ancillary tests and the apnea test, even though they may not be scientifically necessary. Which ancillary test is used depends on the methods available to the clinics. We think that the reason CTA is the most preferred ancillary test in our clinic is its easy accessibility, ease of application and rapid results.

There is no ethical reason to continue medical support to a non-organ donor patient with a confirmed diagnosis of brain death, except in exceptional circumstances, such as when the patient is pregnant. In addition, support of brain dead patients constitutes an unnecessary use of resources and bed occupancy. Escudero et al.^(23)^ reported that it was possible to withdraw medical support and mechanical ventilation from 75% of nondonor patients after brain death. Patients diagnosed with cardiac death and patients diagnosed with brain death are legally equivalent, and the same procedures should be performed. Supportive treatment can be continued if the patient diagnosed with brain death is pregnant or if the relatives of the patient may change their opinion regarding organ donation. In our study, we found that withdrawal of medical support and mechanical ventilation was 35% and that nondonor brain death patients lived on average 2.8 ± 0.3 days. In Turkey, withdrawal of medical support and mechanical ventilation was subject to family approval until 2012, but as of 2012, the approval requirement was removed from the law;^(35)^ however we see that this process is still continuing, albeit partially. There may be legal, cultural, regional and religious reasons for continuing medical support after the diagnosis of brain death. Further research is needed in this field to reveal these reasons.

## CONCLUSION

The diagnosis of brain death is primarily clinical. Ancillary tests should not replace a comprehensive clinical examination. An apnea test is performed in all patients meeting all other brain death criteria who are stable enough to undergo the test. Ancillary tests should be performed on patients when there is difficulty making the diagnosis of brain death in the countries where ancillary testing is not compulsory. It should not be forgotten that there is no worldwide consensus on the use of ancillary tests.
